# Process Evaluation of Project FFAB (Fun Fast Activity Blasts): A Multi-Activity School-Based High-Intensity Interval Training Intervention

**DOI:** 10.3389/fspor.2021.737900

**Published:** 2021-09-20

**Authors:** Kathryn L. Weston, Alison Innerd, Liane B. Azevedo, Susan Bock, Alan M. Batterham

**Affiliations:** ^1^School of Applied Sciences, Edinburgh Napier University, Edinburgh, United Kingdom; ^2^School of Health and Life Sciences, Teesside University, Middlesbrough, United Kingdom; ^3^School of Human and Health Sciences, Huddersfield University, Huddersfield, United Kingdom; ^4^Department of Sport and Exercise Sciences, Durham University, Durham, United Kingdom

**Keywords:** high-intensity, adolescents, school-based, exercise, qualitative

## Abstract

**Introduction:** Over the last decade, research into the impact of school-based high-intensity interval training (HIIT) on young people's health has markedly increased. Despite this, most authors have focused on the outcomes of their intervention, rather than the process of how the study was conducted. The aim of our study, therefore, was to conduct a mixed methods process evaluation of Project FFAB (Fun Fast Activity Blasts), a school-based HIIT intervention for adolescents. The objectives were to explore study recruitment, reach, intervention dose, fidelity, participants' experiences, context, and future implementation.

**Methods:** Recruitment was assessed by comparing the number of students who received study information, to those who provided consent. Reach was described as the number of participants who completed the intervention. Dose was reported via the number of HIIT sessions delivered, total exercise time commitment, HIIT exercise time, and session attendance. Post-intervention focus groups were conducted with intervention participants (*n* = 33; aged 14.1 ± 0.3 years; mean ± standard deviation). These discussions explored aspects of intervention fidelity (extent that the intervention was delivered as intended); participants' experiences of the HIIT sessions; context (exploration of the nuances of school-based HIIT); and ideas for future implementation.

**Results:** Recruitment, reach, and dose data indicate that Project FFAB was largely delivered as planned. Focus group data identified a mismatch between perceived vs. prescribed work: rest ratio for the multi-activity HIIT drills. Generally, the HIIT drills were well-received; participants often reported they were fun to complete, and the use of heart rate monitors was helpful for interpreting exercise intensity. Some participants stated that greater variety in the HIIT drills would be preferable. The timing and structure of the HIIT sessions that took place outside of physical education lessons received mixed responses.

**Conclusion:** Collectively, our study supports the use of school-based HIIT and provides valuable insights into how such interventions can be implemented. Project FFAB could be modified to account for individuals' preferences on when the exercise sessions took place. In addition, a wider range of activities could be included, and the prescribed work: rest ratio of the HIIT drills could be better communicated.

## Introduction

Schools have long been regarded as an ideal public health intervention setting (Kriemler et al., 2011; Dobbins et al., 2013), yet remain busy and unpredictable places (Franks et al., 2007; St Leger et al., 2007) that create challenging conditions for physical activity implementation (van Sluijs and Kriemler, 2016; Bond et al., 2017). Evidence of the effectiveness of school-based physical activity interventions for improving activity levels and/or enhancing health outcomes through exposure is mixed (Kriemler et al., 2011; Dobbins et al., 2013; Love et al., 2019). Recently, a meta-analysis of cluster randomised controlled trials provided strong evidence that school-based interventions do not positively impact on young peoples' daily accelerometer-assessed moderate-to-vigorous physical activity (Love et al., 2019). Therein, however, it was also highlighted that better efforts in the assessment and maximisation of implementation fidelity across such interventions were needed.

Process evaluations are used to monitor and report intervention implementation and help to understand the relationship between intervention content and outcomes (Saunders et al., 2005). A thorough process evaluation can go beyond simply answering whether an intervention “works” from an outcome perspective; by exploring *why* and *how* it was successful (or not), for *who* it could work and how participants react to it (Bauman and Nutbeam, 2013) when implemented in the “real world” setting (Jago et al., 2015; Love et al., 2019). Common features examined in a process evaluation include: (1) recruitment and retention; (2) the number of people who came into contact with the intervention (i.e., reach); (3) quantity of the intervention delivered (i.e., dose) (Moore et al., 2015); (4) how well the intervention was implemented as intended across all participants (i.e., intervention fidelity) (Dumas et al., 2001; Horner et al., 2006); (5) participants' satisfaction, perceptions and usage of the intervention (i.e., participants' experiences) (Moncher and Prinz, 1991; Bellg et al., 2004) and; (6) exploration of how the context affects the implementation of the intervention (Baranowski and Jago, 2005; Bauman and Nutbeam, 2013). Despite the wealth of information process evaluations can offer, surprisingly few appear to be conducted alongside school-based physical activity interventions (Naylor et al., 2015; Love et al., 2019).

Over the last decade, scientific interest in the use of high-intensity interval training (HIIT) as a form of school-based physical activity has markedly increased. Fifteen intervention studies were identified in a 2017 review (Bond et al., 2017), with a 2020 expert statement on the use of HIIT in young people identifying that >10 additional studies have been published since then (Weston et al., 2020). Typically, HIIT is characterised by short, intermittent bursts of very vigorous activity, alternated with periods of rest or low intensity active recovery (Gibala et al., 2012). From a physiological perspective, there is accumulating review-level evidence that HIIT may represent a potent method of improving aspects of cardio-metabolic health and fitness in young people (Logan et al., 2014; Bond et al., 2017; Eddolls et al., 2017; Thivel et al., 2019; Costigan et al., 2015a). However, little is known on exactly how HIIT is implemented as a health intervention outside of the controlled environment of the laboratory. In addition, young people's experiences of taking part in HIIT remains unclear (Biddle and Batterham, 2015; Jung et al., 2015; Leahy et al., 2020; Weston et al., 2020). To date, only three detailed qualitative evaluations of real-world HIIT programmes exist, but these involved adult participants as part of a workplace intervention (Kinnafick et al., 2018; Metcalfe et al., 2020; Burn et al., 2021). Five school-based HIIT studies included aspects of process evaluation within their outcome trials (Buchan et al., 2013; Leahy et al., 2019; Kennedy et al., 2020; Lubans et al., 2020; Costigan et al., 2015b). However, these evaluations were either conducted quantitatively, predominantly focusing on those delivering the intervention (i.e., the schoolteachers) as opposed to those receiving the intervention (i.e., the school pupils) (Costigan et al., 2015b; Leahy et al., 2019; Kennedy et al., 2020; Lubans et al., 2020), or provided inadequate detail about the qualitative methods and analysis techniques utilised (Buchan et al., 2013). These factors limit the in-depth exploration of participants' trial experiences to generate insights and recommendations to guide future research, practise, and policy.

We previously published a quantitative fidelity analysis (Taylor et al., 2015) and outcome evaluation of Project FFAB (Fun Fast Activity Blasts); (Weston et al., 2016), a controlled before-and-after study on the effect of a 10-week school-based HIIT intervention on cardio-metabolic risk markers and physical activity levels in English adolescents. Outcomes assessed at baseline and post-intervention were non-fasting blood lipids and glucose, waist circumference, high sensitivity C-reactive protein, resting blood pressure, daily moderate-to-vigorous physical activity (MVPA), 20-m shuttle-run test performance, and carotid artery intima-media thickness. Post-intervention, we observed favourable changes in blood triglycerides and waist circumference in intervention participants compared to controls. While the outcome evaluation is important from a cardio-metabolic health perspective, crucial aspects of the understanding of intervention functioning have not yet been reported. Therefore, the aim of our current study was to conduct a mixed methods process evaluation of Project FFAB, with the objectives of exploring recruitment, reach, dose, intervention fidelity, participants' experiences, context, and ideas for future implementation. The findings will add to existing evidence on the implementation of the Project FFAB and, more broadly, to the delivery of school-based HIIT interventions.

## Methods

### Ethics Approval and Study Reporting

Ethics approval for all aspects of Project FFAB was obtained from the Teesside University Research Governance and Ethics Committee (study reference 008/11) and the protocol for the outcome trial (Weston et al., 2016) registered on clinicaltrials.gov (trial number NCT02626767). The reporting of qualitative aspects of the present study adheres to the consolidated criteria for reporting qualitative research (COREQ) (Tong et al., 2007). Full details of procedures and results from our previously published work are freely available elsewhere (Taylor et al., 2015; Weston et al., 2016). To contextualise the data collected for the process evaluation, a summary of Project FFAB is provided below.

### Project FFAB Overview

#### Recruitment Procedures

Eight secondary schools in North East England were invited to take part in Project FFAB, based on their geographical proximity to Teesside University, socioeconomic status (assessed through the Index of Multiple Deprivation IMD) (Noble et al., 2008) school specialism and physical education (PE) provision. Schools were initially contacted by emailing the school head teacher and/or head of the PE department. Four agreed; declining schools cited a lack of time and flexibility in their timetable and/or disinterest. All schools were co-educational and provided two 1-h PE lessons per week. To mitigate potential contamination effects between the intervention and control groups (Henaghan et al., 2008), randomisation at the individual level within the schools did not occur. As such, two schools were assigned to the 10-week HIIT intervention (referred to herein as school 1 and 2) and two to the control (school 3 and 4), based on their socioeconomic status and school specialism. According to the IMD ranking, schools 1 and 3 were situated in areas with lower levels of deprivation than schools 2 and 4. At both school 1 and 3, the specialism was sport, whereas at school 2 and 4 the specialism was science and business and enterprise, respectively.

Recruitment took place during PE lessons for Year 9 pupils (aged 13–14 years). Here, potential participants were not made aware of what Project FFAB entailed at other schools, as a means of minimising the risk of bias through contamination issues such as compensatory rivalry or resentful demoralisation (Cook and Campbell, 1979). The target sample size was set at 100 pupils (~25 participants per school), which would inform a future definitive trial by examining whether the intervention could be delivered as intended, with regards to compliance and retention (Craig et al., 2008; Weston et al., 2016). Pupils could participate if they met the health-screening eligibility criteria (Weston et al., 2016) and provided written informed parental consent and participant assent.

#### Project FFAB Intervention

The Project FFAB intervention took place thrice weekly. Two HIIT sessions took place during timetabled PE lessons. The third was conducted after school and during the school lunch break at school 1 and 2, respectively. Following a warm-up, participants performed four repetitions of 45 s of maximal effort exercise (boxing, dance, soccer, and basketball drills) each interspersed with 90-s recovery. All sessions were devised and led by the first author (KLW). The exception was dance activities, which were jointly devised by the first author (KLW) and an experienced dance teacher and led by the latter.

At the start of the PE-based HIIT sessions, participants were fitted with a heart rate monitor (Polar RS400, Polar Electro, Finland). Due to higher numbers at school 2, participants only wore monitors for one PE-based session per week, whereas those at school 1 wore monitors during every PE-based session. To minimise participant burden, heart rate data were not collected at the after school and lunchtime sessions. During HIIT repetitions, participants were verbally motivated to provide “all-out efforts” and if they were wearing a heart rate monitor, to try and reach ≥90% of their individual maximal heart rate on each 45-s repetition (reflecting the high-intensity exercise criterion of previous work (Little et al., 2011). For a full description of the heart rate data collection, reduction, and analysis procedures, please see Taylor et al. (2015) and Weston et al. (2016). Examples of the HIIT drills performed are shown in Table 1. The intervention activities chosen were based on data collected in pre-intervention focus groups with adolescent pupils, which revealed they wanted an intervention which incorporated a variety of activities, with the mode changed frequently (Taylor, 2014). Accordingly, the activities at both schools rotated on a weekly basis.

**Table 1:** Example HIIT drills.

**Activity**	**Drills**
Boxing	Fast jabs on the focus pads Ten jabs on the focus pads, followed by five star jumps Ten jabs on the focus pads, followed by running to the end of the sports hall and back. Ten side steps dodging the focus pads, followed by running to the end of the sports hall and back Five combination punches (hook and jab) on the focus pads, followed by running to the end of the sport hall and back
Basketball	Receiving a returning a chest pass, followed by running to a cone and back Running round in a square and receiving and returning a bounce pass on once corner of the square Bouncing a ball five times then running to the end of the hall and back Receiving a shoulder pass, followed by running to a cone and back
Dance	Jumping up and down whilst waving pom poms above the head Star jumps whilst waving pom poms. Stationary high-knees runs Fast side kicks High leg kicks whilst clapping pom poms underneath the lifted leg
Soccer	Kicking a football into a goal then running to end of the sports hall and back. Ten toe touches, followed by running to a cone and back. Performing fast feet movements through cones then running to end of the sport hall and back Jumping up to head a football five times then running to the end of the sports hall and back Running around the sports hall following a square or diagonal course

At school 1, 17 pupils (9 boys) volunteered to take part in the study. Here, participants attended HIIT sessions as two groups (group 1; *n* = 9 [6 boys] and group 2; *n* = 8 [3 boys]), based on when their PE lessons were timetabled. The session activity mode was chosen collectively by the group. If the decision was not unanimous the small group numbers per session allowed two activities to run concurrently. At school 2, 24 boys provided consent and attended sessions as one group. Here, participants chose between boxing and soccer. Due to the larger group size (*n* = 24), it was only feasible to run one activity per session.

Across the 10-week intervention the number of repetitions performed during each session increased from four to seven. Participants were encouraged to attend as many sessions as possible; those who completed ≥70% were awarded a t-shirt, with individuals attending ≥90% also entered into a prize draw to win a pair of training shoes.

### Process Evaluation

#### Quantitative Data

Recruitment data were collected by recording the number of pupils who received information about Project FFAB and comparing this to the number that provided full informed consent and participant's assent for participation. This figure is reported in raw units and as a percentage. Study reach is described as the number of intervention participants who completed the 10-week intervention programme. For study dose, the number of HIIT repetitions delivered across the 30 sessions scheduled at each school was recorded, along with any deviations in the session scheduling. The total exercise time commitment (inclusive of warm-up and cool-down activities) and the amount of time dedicated to HIIT activities is described in minutes and seconds. Session attendance was recorded via a register at each session and reported as a percentage of the total sessions available (*n* = 30).

#### Qualitative Data Collection: Post-intervention Focus Groups

##### Participants and Recruitment

Within 3 days of the final HIIT session, all participants who completed the 10-week programme and/or the post-intervention outcome measures data collection (*n* = 40) were invited to take part in a focus group to discuss their experiences of the intervention. In total, 33 students (aged 14.1 ± 0.3 years) provided parental consent and participant assent. Reasons for non-participation were forgetting to return parental consent forms (*n* = 5) and electing not to take part (*n* = 2).

##### Protocol

Five focus groups took place across the two schools. At school 1, two focus groups consisting of eight (three girls) and six (three girls) participants were conducted. Three focus groups took place at school 2. The first was attended by seven boys and the second and third by six boys each. As the HIIT sessions were group-based, we adopted a semi-structured focus group design to reflect this dynamic and to facilitate discussion amongst the participants (Horner, 2000).

The focus group script (Supplementary File 1) was primarily devised by the first author (KLW); then shared with two authors (AI, AMB) for feedback. Discussion topics were structured around the following process evaluation elements: intervention fidelity (the extent to which the intervention was delivered as intended); participants' experiences of the intervention (participants' thoughts on the content, structure, delivery, and timing of the HIIT sessions, and facilitators and barriers to programme adherence and attendance); context (exploration of the nuances of school-based HIIT; and future implementation (whether similar HIIT programmes could, should or should not be delivered in the future) (Glasgow et al., 1999).

Focus group sessions were led by the second author (AI) who was a female postgraduate researcher with both positive and negative experiences of participating in HIIT, 3 years of qualitative research methods experience and who had received post-graduate level training in qualitative research methods. She had not been present at the HIIT sessions, but the participants were familiar with her from baseline data collection. The focus groups took place in classrooms during participants' PE lessons, with the researcher sitting amongst the participants. All sessions were recorded using a digital recorder (Edirol R-09HR; Roland).

##### Data Analysis

The focus group recordings were transcribed verbatim and anonymised by the first author (KLW) and checked for completeness and accuracy by the third author (LA). There were 75 pages of raw transcription data (Arial font size 12, single line spacing). In line with previous guidance on thematic analysis (Braun and Clarke, 2006), the following steps were conducted. All transcripts were read and re-read to enable familiarisation with the data. Transcripts were then re-read line by line and marked with initial codes that described the content. To maximise consistency and completeness, these steps were completed by three of the authors (KLW, AI, LA). Following discussions on the initial coding process, the second author (AI) deductively developed themes within the data and drafted initial data themes. All authors then reviewed and refined the themes using an iterative process, until agreement on the themes was reached and the final themes named. Finally, the themes were presented in a coherent and logical way alongside quotes deemed to best illustrate each theme (Braun and Clarke, 2006). Given recent critiques of the concept of data saturation (Braun and Clarke, 2019; Low, 2019), we did not seek to assess data saturation.

## Results

### Quantitative Data

#### Recruitment and Reach

During recruitment, PE teachers at school 1 elected to exclude pupils in the highest ability PE classes due to examination commitments. Consequently, the study was introduced to 80 male and female pupils, of which 17 (9 boys; 21% recruitment rate) volunteered. At school 2, the study was introduced to 30 male students, 24 of whom provided consent (80% recruitment rate). At the control schools, Project FFAB was introduced to 45 and 30 pupils, respectively, of which 37 (22 boys; 82% recruitment rate) and 23 (8 boys; 77% recruitment rate) consented. The total sample size at baseline was 101 (62 boys; mean age ± standard deviation [SD] 14.0 ± 0.3 years) (55% overall recruitment rate), of which 41 (aged 14.1 ± 0.3 years; 33 boys) attended intervention schools. For further detailed descriptive data on the study population, please see Weston et al. (2016).

Of the 41 participants who began the 10-week intervention, 38 completed. Two boys sustained injuries unrelated to the study after weeks 5 and 7, and therefore did not complete the remaining HIIT sessions but were available for post-intervention testing and attended the focus groups. No injuries related to the intervention were reported. One girl dropped out after week 6 citing a lack of interest and had no further involvement with Project FFAB.

#### Dose

Across the intervention, 159 HIIT repetitions were delivered across 30 sessions at each intervention school. Two sessions at school 2 had to be cancelled due to school events replacing PE lessons; however, these were rescheduled as additional lunch time sessions, thus still allowing all 30 planned sessions to take place. The total exercise time commitment was 419 min and 15 s, inclusive of warm-up and cool-down activities. The amount of time dedicated to high intensity activities therefore was 119 min 15 s (~12 min per week). Mean (SD) attendance (expressed as a percentage of total sessions) was 77%. For a detailed analysis of heart rate data collected during the intervention, please see Taylor et al. (2015) and Weston et al. (2016) Reasons for individual deviations from the exercise dose can be found in the qualitative data sections.

### Qualitative Focus Group Data

We combined qualitative data from 33 participants across five focus groups and identified themes which related to the process evaluation components of: (1) intervention fidelity; (2) participants' experiences; (3) context; and (4) future implementation. Within the illustrative quotes, participants refer to soccer as “football” and all mentions of “she” refers to the HIIT session instructor and first author (KLW), unless otherwise stated.

#### Intervention Fidelity

**(A) HIIT Sessions: Awareness of Work: Rest Ratio**
  When discussing what the HIIT sessions entailed, participants spoke about the length of the repetitions and the subsequent rest period. Generally, participants thought the rest interval was equal to, or less than the length of the HIIT repetition.
  “*You got 45 seconds to do it, and then 45 seconds rest before you move onto the next one.”*
  (Female participant, school 1, focus group 2)
  “*The rest seemed shorter than the running….I think she was trying to fool us. To make us work harder.”*
  (Male participant, school 2, focus group 4)
  “*I think there could have been like, a bit more timing between activities. You know like to catch your breath. After every activity like* (there should be)…* About a minute, minute and a half…A minute or something to catch our breaths.”*
  Male participant, school 2, group 5
  Some participants thought the time in between intervals and the number of rest periods was too little for them to recover.
  “*Like when people like were standing and out of breath because she was pushing us too fast, like she was still telling us to go on but we were still like, really out of breath so it was very hard.”*
  (Male participant, school 2, focus group 5)
  “*It was good like, but we never had that many breaks so you couldn't get your breath back.”*
(Male participant, school 2, focus group 3).
**(B) HIIT Sessions: Awareness of Exercise Intensity**
  Across the focus groups, participants said they were asked to try hard during the HIIT repetitions. The role of the heart rate monitors was often discussed as a means of gauging how hard they were working, and participants frequently reported that a high value on their heart rate monitor indicated they were working at the desired intensity.
  “*Because your heart rate* (monitor), *it tells you what your heart rate was and like, if it was high then you* (had) *tried really hard and if it was low, you weren't really working.”*
  (Male participant, school 2, focus group 3).
  “*Yeah, and we were wearing heart monitors, like if you'd got to 190* (beats per minute [bpm]) *or even over 200* (bpm) *you were doing quite well.”*
  (Male participant, school 2, focus group 5)
  “*And eh, ‘cause of the things that you had on your arm, she said like, try to get this certain, eh heart rate, and if you didn't then you'd know to try harder next time. After a few weeks I'd, worked out what I could do. I realised that* (at) *about 180 to 190* (bpm), *I was trying, but I could try harder…Once I got to 200* (bpm)…*that was like, I was trying really hard.”*
  (Male participant, school 1, focus group 1)
**(C) HIIT Sessions: Deviations From Intended Exercise Intensity**
  When discussing HIIT session intensity, participants often compared the PE-based sessions to the after school or lunch time ones. Some participants thought the intensity across all was relatively uniform. Others thought more effort was put into the PE-based sessions.
  “*The ones after school were a bit easier because eh, ‘cause you didn't have your heart monitor thing on, you sort of didn't realise when you were doing well and when you weren't, so it was a bit easier… like you could do it easier than trying flat out.”*
  (Male participant, school 1, focus group 1)
  “*The Friday lunchtime one always seemed easier than the ones that we did in P.E. Like not as many people like took the Friday session serious, like when they were in P.E. they took it really serious but like when it wasn't P.E they were just messing around and that”*.
  (Male participant, school 2, focus group 4)
  Some participants at school 2 reported that they put in less effort toward the end of the intervention as they got used to the activities.
  “*We got used to what we were doing… Yeah because, when people were getting used to it, they just didn't try because it was just the same. Yeah, they were used to it every week and they thought if like we tried last week, why should we try again this week?”*
  (Male participant, school 2, focus group 4)
**(D) HIIT Sessions: Reasons for Deviations From Individuals' Exercise Dose**
  Generally, the PE-based sessions were well-attended across both schools, with participants only missing sessions if they were unwell or on holiday. The timing of the after-school session at school 1 sometimes left participants unable to attend due to other commitments.
  “*I didn't go to any of the after* (school sessions)*, on Thursday after school because at first I'd got like family things and then I had to go to my guitar lesson on Thursday night.”*
  (Male participant, school 1, focus group 1)
  “*I missed a few (*after school*) sessions with auditions and rehearsals and stuff which I couldn't miss.”*
  (Male participant, school 1, focus group 1).
  At school 2, participants reported that fewer people attended the lunchtime sessions.
  (At) “*the lunchtime one, barely anyone showed up and some people only showed up if they felt like it or they were bored.”*
  (Male participant, school 2, focus group 5)
  “*I preferred them at lunchtime because there's less people there so there was less people messing about, so you do it quicker”*.
  (Male participant, school 2, focus group 3).

#### Participants' Experiences

**(A) Physical Responses to Performing HIIT**
  Participants often reported feelings of tiredness after performing the HIIT repetitions and spoke about the bodily cues which indicated this.
  “*Your body like sort of tells you* (how you are doing) *seeing as like, you're out of breath”*
  (Male participant, school 1, focus group 3)
  “*It depends which one you did, like the boxing one, it would like make your arms ache a bit but it would be alright, not that bad but like with the footballing you'd be more out of breath….The boxing one was like more on your muscles and stuff and football is like more stamina and stuff”*
  (Male participant, school 2, focus group 4)
**(B) Psychological/Affective Responses to Performing HIIT**
  Some participants spoke about the psychological feelings and emotions they experienced during the sessions.
  “*Like, at the end of it I felt quite good ‘cause like, I'd done the exercise, I didn't think aw I'm shattered, and I thought like, I'm tired but it's good.”*
  *(*Male participant, school 1, focus group 1)
  “*It gives you a buzz.”*
  (Male participant, school 2, focus group 3)
  “*Made me feel happy…. Like sort of a rush.”*
  (Male participant, school 2, focus group 3).
**(C) HIIT Modes**
  When recalling what was done during the HIIT sessions, participants discussed their personal likes and dislikes. Across the groups, no one activity emerged as the most popular.
  (I liked best) “*The running, just like the square run thing, then basketball, then football, then eh the dance…. I didn't like the dancing ‘cause it was a bit girly.”*
  (Male participant, school 1, focus group 1)
  “*I liked it all, but I've gotta say dancing ‘cause I really like dancing… Then cheerleading ‘cause I'd always wanted to do it and it was something I never got round to doing until I did it here. And then the boxercise ‘cause it was really fun. I was like, really lazy, but once we started challenging the others I got really competitive. And then last the running cause I'm not really a running person.”*
  (Female participant, school 1, focus group 1).
  Often, participants explained that their reason for liking an activity was because it was fun and/or something different.
  (I liked) “*none of them, they're all too much exercise* (laughs)…* No probably the dance, cause, it was just fun”*
  (Male participant, school 1, focus group 2)
  “*I just liked it. It was different from what you normally do in football, in a PE lesson… We did that toe touching thing that I've actually never done before and I actually started to get better at it.”*
  (Male participant, school 1, focus group 2)
  “*The boxing one because it's like you laugh and stuff whilst you're doing it, do you know what I mean? Not messing about but you can have a laugh while you're doing it.”*
  (Male participant, school 2, focus group 4)
  Across the focus groups, some of the running activities incorporated into the soccer and basketball drills were not viewed favourably.
  “*I don't really like big, when you have to do the big run around right at the end, that makes you absolutely knackered. Like when you run round the square.”*
  (Male participant, school 2, focus group 2)
  “*I don't like the one…when you have to do the cross* (across the sports hall) *in between the run. It was like, really confusing”*.
  (Male participant, school 1, focus group 2)
  At school 2, participants reported that in the later weeks of the intervention, repetition of the HIIT activities led to boredom and a lack of enjoyment.
  “*I wouldn't change it except for like the last couple of weeks because it was kind of just like it got so repetitive we just kind of guessed what was coming….we could have done the lessons by ourselves, we wouldn't have needed anyone there so it kind of took the enjoyment out of it”*.
  (Male participant, school 2, focus group 4)
  “*Like* (we could have included) *different sports like rugby and cricket. Because we all tended to like football or boxing so it was all a bit too repetitive.”*
  (Male participant, school 2, focus group 4)
  “*They* (the HIIT sessions) *were good but.Because everyone else was talking so, she had it all planned out, she had it all written but she couldn't do anything because we were all talking, during the later sessions. At the beginning we didn't, most of us were talking towards the end…. because we got bored.”*
  (Male participant, school 2, focus group 3)
**(D) HIIT Session Timing**
  At school 1, some participants preferred the timing of the non-PE based session, as it enabled them to work in a different way than during the PE-based sessions.
  “*Yeah, ‘cause* (at the after school sessions) *you only get like 20 minutes to do it, so it's like you push yourself.”*
  (Male participant, school 1, focus group 2)
  At school 2, participants generally viewed the timing of the non-PE based session negatively, with many perceiving the sessions took up too much of the school lunch break.
  “*I think for some people it was the idea of losing their dinnertime because like everyone messed around in the* (lunch time) *session so you got less time to do it and then… then you lost a lot of your lunch.”*
  (Male participant, school 2, focus group 4)
  “*Yeah, because no one wanted to do it* (lunchtime sessions)…*.Because lunchtime is for socialising.”*
  (Male participant, school 2, focus group 3)
**(E) Instructor Delivery**
  Across the groups, the instructors' engagement and interactions with the participants was viewed positively, with participants often noting the verbal encouragement they received helped them during the HIIT sessions.
  “*I felt yeah, that she pushed us and did good…. That made me want to go faster to impress her more.”*
  (Male participant, school 2, focus group 3)
  “*She was good, like constantly encouraging you to keep going… She'd join in if she needed to.”*
  (Male participant, school 1, focus group 1)
  “*The dance instructor, she was proper encouraging everybody. She was like come on, get up, get up, come on!”*
  (Female participant, school 1, focus group 2).
**(F) Perceptions of personal change**
  Often, participants spoke about how they performed over the course of the intervention.
  “*As the weeks went on you improved on certain things… Like when you started off you'd just be like oh right this is what we're doing sort of thing, and then after the weeks, you sort of like, learnt how to do them better and you could do them more easily. The day after* (cheerleading), *I mean, I know me and* (another participant) *were cause we're in the same form, but our legs were absolutely killing. But weeks, like weeks later when we done it, it didn't hurt as much, so fitness definitely improved for me.”*
  (Male participant, school 1, focus group 2)
  “*We knew what we were going to do and we were ready for it… And I think it helped you.”* (Male participant, school 2, focus group 4)
  Across the groups, participants spoke about how they perceived they had changed following the intervention. Some reported feeling the same as they did before the intervention. Participants often discussed how they thought aspects of their physical fitness had improved.
  “*I feel stronger and faster than I was at the start of this thing”*.
  (Male participant, school 2, focus group 5)
  “*When I did the school fun run thing, like last time* (before the intervention) *I came quite far behind but this time I just kept running and I had more stamina.”*
  (Male participant, school 1, focus group 1)
  “*It's like energetic or whatever, like takes a lot of energy away but like you feel better about it at the end, like once you've done it you realise it was like, it helped your fitness.”*
  (Male participant, school 2, focus group 5).
  “*You can have like, fun, but get fit at the same time.”*
  (Female participant, school 1, focus group 2)
  Some participants spoke about psychological changes they experienced following the programme.
  “*It gave me a lot more thought of what activities I can actually try out and what I like…. Yeah and what I can do, and that I can actually try if I really want to”*
  (Female participant, school 1, focus group 1)
  (Feel) “*Happy because you got something out of it. Learning new like ways to exercise”*.
  (Female participant, school 1, focus group 2)
  “*You're like, more confident.”*
  (Male participant, school 2, focus group 3)
**(G) Incentives**
  The role of physical incentives was rarely discussed at school 1. At school 2, participants frequently discussed the incentives they would receive following the completion of the programme. Here, the inclusion of incentives was viewed positively and cited as a reason to attend sessions. Some participants also suggested that they would not do a similar programme in the future if incentives were not offered.
  “*Towards the end, she said some of you are doing quite well, you've passed 90% (*attendance). *And* (at) *over 90% attendance you get put in for a draw for some trainers.… We wanted the trainers.”*
  (Male participant, school 2, focus group 5)
  “*If you get 70%* (attendance) *you get like a branded T-shirt and shorts or something”*
  (Male participant, school 2, focus group 3)
  “*Do we get rewards or do we not get rewards?* (if it was continued) *Because if there's no prizes there's no point sticking in with it if there's no prizes.”*
  (Male participant, school 2, focus group 3).

#### Context

**(A) Role of PE for Delivering HIIT**
  Using the PE lesson as a vehicle for delivering HIIT was discussed in a variety of ways across the groups. At school 1, the HIIT intervention was compared to normal PE lessons.
  “*Yeah…you definitely get to do something in this* (Project FFAB)…*In PE you don't necessarily do anything, you can just be like standing around doing nothing but in this, like you have to like get up and do something. It's actually, you know, doing something”*.
  (Male participant, school 1, focus group 2)
  “*You only like stand around in PE and just get like one go of like batting the ball and then you…. And then you sit down. ‘Cause everybody else is like dead lazy.”*
  (Female participant, school 1, focus group 2)
  At school 2, participants discussed that by taking part in Project FFAB, it had impacted on parts of their normal PE curriculum. Generally, this was viewed negatively.
  “*We missed most of our athletics because of this.”*
  (Male participant, school 2, focus group 3).
  (I'm)“*Glad (*the project is finished). *Because now we don't have to do anymore of this, even though it was good but like, it does honestly get tiring after like so many weeks, and we get prizes. And plus we can get on with our regular stuff because around about now we'd be doing tennis and softball, wouldn't we?”*
  (Male participant, school 2, focus group 3).

#### Future Implementation

**(A) Use of PE Time for HIIT Delivery**
  At school 2, whether future HIIT sessions could be delivered as part of normal PE lessons received mixed responses.
  (Would) “*Like* (it in) *PE lesson time. Yeah, because you're already like ready for lessons so you might as well do it…Yeah, not in like our personal time.”*
  (Male participant, school 2, focus group 4)
  “*I prefer it in the PE class because like when you go and you're working with your friends.”*
  (Male participant, school 2, focus group 3)
  (Not as part of PE lesson) “*Then you can choose whether you want to do it or not.”*
  (Male participant, school 1, focus group 2)
**(B) Use of Non-PE time for HIIT delivery**
  Across the groups, participants discussed different options relating to whether HIIT could be delivered outside of PE lessons and/or outside of the school setting. Several options were suggested but no single one emerged as the most popular or practical.
  “*I do tonnes of clubs so after schools ones weren't very good ‘cause I'd like miss them sometimes, and on a weekend I'm really busy so I think the best time would be at lunchtimes ‘cause everybody's at school anyway and all you usually do is like, eat your lunch*.
  (Male participant, school 1, focus group 1)
  “*Em, it depends, it depends what days it's on, like Saturdays and Sundays no, ‘cause like that's the time we chill and like just hang out with our mates. Mondays and Wednesdays people have stuff on, I'd probably do it Friday afternoon because that's when school's finished, like you've got nothing else to do, all you do is just sit around doing nothing and watch telly. Like it has to be very close as well like, say it could be in… just in different places and different people could run it.”*
  (Male participant, school 2, focus group 5).
  “*On the weekends ‘cause more people are free and more people will be bored on a Sunday ‘cause more people will be bored, and the more people will turn up”*.
  (Female participant, school 1, focus group 1).
**(C) Future Recruitment and Retention Strategies**
  Lastly, participants discussed how future interventions could be delivered to encourage recruitment and retention. Frequently, participants suggested increasing the rest interval of the HIIT repetitions.
  “*To make it more appealing for like lazy people - slow it down a bit*, (which would) *give you like longer time for rests and longer time to do the activities instead of like going round at like 700 miles per hour in like 45 seconds running, get like 90 seconds”*
  (Male participant, school 2, focus group 3)
  “*Get a bit of a longer break, ‘cause like you've only just calmed down and you have to do like another exercise straight away.”*
  (Female participant, school 1, focus group 2)
  Participants also suggested more focus should be placed on the HIIT activities, and less on the outcome measures.
  “*Do like advertise more on the like, the activities rather than like the tests and stuff that you do”*
  (Female participant, school 1, focus group 1)

## Discussion

Despite increasing research interest in the use of school-based HIIT as a means of improving health and well-being outcomes in young people (Weston et al., 2020), the process of implementing such interventions, and young people's experiences of HIIT programmes, are underreported. Accordingly, the aim of our study was to conduct a process evaluation of Project FFAB, a multi-activity school-based HIIT intervention for English adolescents. By utilising a mixed methods study design, we were able to combine traditional process evaluation approaches, including quantitative assessments of recruitment, reach, and intervention dose, with in-depth qualitative accounts from the intervention participants on intervention fidelity, their HIIT experiences, context, and future implementation.

Across Project FFAB, the target sample size was 100 participants. Collectively, this number was achieved across the four intervention and control sites, where 101 participants were recruited at baseline and only one intervention participant dropped out during the study. At school 1 however, only 17 of 80 invited pupils volunteered to take part in Project FFAB (21% recruitment rate compared to the four-school average of 55%). This can possibly be explained by the PE teacher's decision to exclude students from the highest ability PE class in school 1 from volunteering, as it has been suggested that HIIT may appeal most to those who are already active (Biddle and Batterham, 2015). By excluding pupils who may reflect this demographic, overall recruitment may be lower. From a logistics perspective, the recruitment rate may also have been negatively impacted by the way the study was introduced at school 1. At school 2 and both control schools, the study was introduced to separate classes of a maximum of 45 pupils (range 30–45), which allowed for individual questions to be answered in real time. At school 1 however, the study was introduced to the whole Year 9 group (80 pupils) at once, which did not allow for the same level of interaction between potential participants and the researcher.

With regards to the intervention dose, descriptive data presented here and elsewhere (Weston et al., 2016) indicate that Project FFAB was delivered as intended, in terms of the number of HIIT sessions provided (*n* = 30). However, while overall session attendance was high (77%), the focus groups revealed factors that influenced individual participants' HIIT dose across the intervention. At school 1, after-school sessions were sometimes missed due to clashes with other extra-curricular commitments. At school 2, participants observed that fewer people attended the lunchtime sessions than the sessions delivered during PE. This could be attributed to an overall sense that the purpose of the school lunch-break is to spend time with friends and eat lunch, rather than perform structured exercise.

In the context of the fidelity of the intervention, the focus groups highlighted a discrepancy between what participants perceived the HIIT work: rest ratio to be, and what it actually was. As detailed in the methods, each HIIT repetition consisted of 45-s of activity, followed by 90-s recovery (i.e., work: rest ratio of 1:2). Participants however, reported that the rest interval was equal to or less than the length of the HIIT interval. This was generally viewed negatively and brought up when potential changes to future programmes were discussed. This misconception is concerning as it could lead to participants becoming less engaged or motivated to participate in the sessions, which could impact on study retention, fidelity, and future physical activity behaviours. One practical way of addressing this confusion is to provide a visible or audible timer for participants to access during the HIIT sessions, which would allow the length of the work and rest intervals to be clearly communicated. Alternatively, future studies could explore manipulating the work: rest ratio of the HIIT sessions (e.g., altering the work: rest ratio and/or relative intensity of the individual repetitions), as a means of maximising participants' enjoyment and engagement. Indeed, there is evidence to suggest that varying exercise intensity, duration, and work: rest ratios within a HIIT session can impact affect, perceptions of effort, and enjoyment (Martinez et al., 2015; Malik et al., 2019). It is therefore possible that a more personalised/individualised approach is needed to titrate the appropriate dose of HIIT with respect to work: rest ratio, as a means of preserving physiological benefit while minimising negative affective responses. While this interesting avenue of work has begun to be explored in adolescents from an acute perspective in the laboratory setting (Malik et al., 2019), it has not yet been applied in the school environment.

When describing the intensity of the HIIT repetitions, participants frequently mentioned the use of heart rate monitors as a means of gauging how hard they were working. Generally, the participants viewed the heart rate monitors as a helpful way of gaining feedback and displayed a good awareness of heart rate values indicative of high-intensity work. This was despite never being explicitly told what 90% of their individual maximal heart rate corresponded to. These findings highlight the importance of personalised feedback (Turk et al., 2013; Arambepola et al., 2021) during HIIT, and the wider potential of wearable technology in engaging people in physical activity and exercise (Ridgers et al., 2016). Nonetheless, while the use of wearable technology in the PE setting is being explored (Marttinen et al., 2019), it is imperative to acknowledge the cost associated with such devices. Indeed, while an individual heart rate monitor would provide an objective means of assessing HIIT exercise intensity, it is highly questionable whether research and/or school budgets could accommodate this requirement for a scaled-up, multi-site intervention. This highlights the trade-off that is often apparent in applied research, between gold standard measurement and pragmatic alternatives. While other school-based HIIT studies have utilised tools such as Session Rating of Perceived Exertion as a way of prescribing exercise intensity (Bond et al., 2017), our findings suggest this may be inadequate for communicating and monitoring exercise intensity in young people who may be unaccustomed to performing high-intensity activity. Nonetheless, our participants did mention several bodily cues they thought were indicative of high-intensity work, including feeling out of breath and local muscular fatigue. If cost and time negated the use of objective heart rate monitoring, a potential alternative is the use of differential ratings of perceived exertion scales, which allows discrimination between central (i.e., breathlessness) and peripheral (i.e., arms and legs) exertion (McLaren et al., 2016).

In our previously published quantitative fidelity analysis of Project FFAB, we suggest that the fidelity of our intervention was moderate at best (Taylor et al., 2015). This conclusion was largely based on the analysis of intervention heart rate data which revealed variability in the intensity of the HIIT sessions between and within participants. Data from the focus groups provides a deeper understanding on why these variations may have occurred. For example, across both intervention schools the non-PE based sessions were viewed as easier and less well-structured than the PE-based ones. This perception could have been partly due to the lack of heart rate monitoring at the non-PE sessions, which may have left participants less able to gauge the intensity they were working at. As a third of the total intervention consisted of non-PE based sessions (i.e., 10/30), this perceived lack of consistency could have negatively impacted study outcomes where changes are driven by exercise intensity. One such example is 20 m shuttle run test performance, which did not substantially improve post-intervention (Weston et al., 2016). This finding is at odds with that reported in other school-based HIIT trials (Bond et al., 2017; Eddolls et al., 2017), yet may now be partly explained by observations that HIIT was performed at a lower intensity at the after school and lunchtime sessions. One potential way of addressing this is to schedule all HIIT sessions during PE curriculum time, since these sessions were generally viewed more positively in terms of structure and intensity. This approach could, however, take away from other valuable aspects of the PE lesson from a pedagogical perspective, and may not be well-received by PE staff or pupils. In our participants, views on continuing HIIT style activities in PE lessons beyond Project FFAB were very mixed, which demonstrates the complexity of HIIT programming for a diverse group within the constraints of the school timetable.

With regards to participants' experiences of the different HIIT modes utilised, no single activity clearly emerged as the least or most popular. Generally, participants spoke negatively about drills they perceived to be too complicated or hard, such as some of the running activities. Overall, these findings are unsurprising as Project FFAB was designed to provide a variety in HIIT modalities (e.g., boxing, dance and soccer) from the outset, based on formative evaluation data collected in the development stage of the intervention (Taylor, 2014). Midway through the intervention, basketball drills were added to the HIIT options at school 1, following observations that participants engaged well with simple ball-based activities and spoke enthusiastically about basketball.

Due to the smaller group sizes at school 1, it was possible for at least two HIIT activities to run concurrently during a single session. At school 2 however, only boxing and soccer drills were performed, owing to disinterest in other activities suggested by the first author, a lack of appropriate equipment (e.g., basketballs), and the large group size (24 participants). Here, participants were also sometimes prone to chatting and misbehaving, especially in the later stages of the intervention as noted in their focus groups. Such issues have been acknowledged previously, with warnings that unruly and overly demanding participants can disrupt group sessions, leading to a lack of participation by others (Orwin, 2000). At school 2, participants complained they had become bored with the repetitive nature of the sessions by the end of Project FFAB, which highlights the need to continually refresh activities across an intervention where possible (Taylor et al., 2018; Innerd et al., 2019; Jong et al., 2020). Further, it is acknowledged that participants at school 2 did not receive the autonomy and same degree of variety that participants at school 1 received, which may have negatively impacted on their enjoyment and engagement with the later HIIT sessions.

Participants also shared why they liked or disliked certain HIIT activities. Often, they stated they liked a specific activity because it was fun. Since fun and enjoyment are often cited as the most important factors for engaging young people in physical activity and positive health behaviours (van Sluijs and Kriemler, 2016; Strömmer et al., 2021), this finding may illustrate the potential of novel HIIT modes to engage young people in sport, exercise, and physical activity. The finding also partly alleviates concerns expressed in the literature (Biddle and Batterham, 2015) which suggest performing HIIT will evoke feelings of displeasure (Ekkekakis, 2003) and could negatively impact on future exercise behaviour (Rhodes and Kates, 2015). While we did not collect acute or chronic objective measures of exercise affect, enjoyment, or psychological well-being during Project FFAB, data from our focus groups provide some evidence of positive feelings after the individual HIIT repetitions and post-intervention and elaborate on why this may have been the case. Given the importance of young people's mental health and well-being, exploring these responses in the long-term is a key area for future research. While preliminary data from three school-based HIIT interventions in older adolescents suggests that school-based HIIT may improve some aspects of mental health (e.g., increases in executive function and reductions in psychological difficulties) (Costigan et al., 2016; Leahy et al., 2019; Lubans et al., 2020); these data can only be considered as pilot due to small and homogeneous samples. Further, while some initial data suggest that adolescents may enjoy acute bouts of HIIT, these data have been collected in a laboratory setting (Malik et al., 2018), which does not mirror the often group-based environment of school-based HIIT protocols.

When reflecting on their overall experience of Project FFAB, some participants felt aspects of their physical fitness improved post-intervention. This finding is interesting, given that no substantial mean changes were observed in the intervention group for 20 m shuttle run test performance compared to the controls (Weston et al., 2016). While this may reflect individual variability in participants' response to the intervention, it is also possible that other components of physical fitness, such as muscular power and sprint ability, improved but went undetected since a broader physical fitness testing battery was not implemented. In light of this, we recommend that future HIIT trials look beyond the intervention effects on cardiorespiratory function alone and include measures of muscular fitness and sprint ability in their physical fitness testing battery (Buchan et al., 2011, 2013).

At school 2, participants often spoke about the role played by the incentive of winning prizes at the end of Project FFAB on their continued participation in the HIIT sessions. Largely, the incentives were viewed positively, with some participants reporting they would not have engaged with the project otherwise. As the role of incentives was rarely mentioned at school 1, this could highlight a difference in participants' intrinsic and extrinsic motivation for remaining involved in Project FFAB (Deci and Ryan, 2000). While the concept of providing incentives for positive health behaviours is widely debated in the literature (Mitchell et al., 2020), from a cost and scalability perspective, it is highly unlikely that continually providing rewards for engagement in HIIT activities would be sustainable in the long-term. This issue raises interesting questions on whether it is possible to alter an individual's motivation to perform HIIT from extrinsic to intrinsic factors. Though beyond the scope of the current study, this question highlights a complex and much needed area for future research on long term engagement with HIIT behaviours. Given that school 2 was located in a geographical area with higher levels of deprivation than school 1, it is possible that the contrasting importance of incentives may be partly explained by socioeconomic differences. Indeed, research suggests that those from a higher socioeconomic status area (i.e., school 1 participants) are more enthusiastic and aware of the benefits of leading healthy lifestyle (Hanson and Chen, 2007) which may be reflected in our study.

From our findings on participants' experiences, study context, and future implementation, it is clear no one-size-fits-all strategy exists for school-based HIIT, and implementation can be further complicated by constraints within the school day and calendar. As expected, participants' enjoyment and engagement varied depending on the HIIT activity, so it remains crucial that future HIIT interventions for young people try to closely replicate young peoples' physical activity preferences and patterns (Weston et al., 2020). The role of the HIIT deliverer also appears to be important for ensuring a positive HIIT experience in young people. In our study, participants viewed the encouragement they received from the instructors as helpful, which should be replicated in future work. Consideration to intensity monitoring (e.g., heart rate monitor) also appears to be key in engaging participants and maximising intervention fidelity. From a programming perspective, Project FFAB could be improved by regularly refreshing the HIIT activity options throughout the intervention to minimise boredom, ensuring the participants are aware of exactly how long the work: rest ratio is, and exploring the option of non-PE based sessions in consultation with the PE staff and prospective participants. The notion of HIIT sessions only taking place during PE remains contentious, unless a scalable model where HIIT is performed in addition, as opposed to in replacement of PE activities can be developed. Early success in this regard has been shown in the “Burn2Learn” intervention for older adolescents in Australia, where HIIT sessions are teacher- as opposed to researcher-led (Costigan et al., 2015b; Kennedy et al., 2020; Lubans et al., 2020).

In terms of outcome measures, future studies should continue to explore the role of school-based HIIT in young people's mental health, and health outcomes beyond the traditional cardio-metabolic measures assessed to date. When considering the strengths of our current study, it is important to highlight that our focus groups involved >80% of the participants who took part in the intervention, which maximises the likelihood that the data collected were representative of the intervention group as a whole. We have also detailed in-depth adolescent experiences of school-based HIIT for the first time. Where possible, future process evaluations of school-based HIIT should incorporate both teacher and pupil perspectives. The former was not the focus of our study, however by involving teachers in future evaluations questions on the scalability of projects could be better answered. As we did not conduct focus groups in the control schools, adolescent perceptions of being part of a school-based programme in general remain unknown. This could also be explored in future work.

## Conclusions

Collectively, this mixed methods process evaluation of Project FFAB offers rich insights into how a HIIT intervention can be implemented within the school setting. By interviewing participants involved in Project FFAB, we have given a voice to young people in the research process, who have largely been overlooked previously. Overall, the multi-activity HIIT drills were well-received, implemented as planned, and participants often reported they were fun to complete. The programme could be modified to account for individuals' preferences when non-PE based sessions took place, include a wider range of activities, and communication of the work: rest ratio could be strengthened. While not assessed objectively, participants reported some positive physical and psychological benefits beyond the scope of the study, which should be explored in future work. Overall, the study findings support the use of school-based HIIT as a means of engaging young people in physical activity, and provides valuable insights into how HIIT can be implemented in community settings.

## Data Availability Statement

The raw data supporting the conclusions of this article will be made available by the authors, without undue reservation.

## Ethics Statement

The studies involving human participants were reviewed and approved by Teesside University Research Governance and Ethics Committee. Written informed consent to participate in this study was provided by the participants' legal guardian/next of kin and written assent was obtained from the study participants.

## Author Contributions

KW, AI, and AB designed the study. KW, AI, and LA completed data collection and performed the data analysis. KW wrote the first draft of the manuscript. All authors contributed to manuscript revision, read, and approved the submitted version.

## Funding

The work was undertaken as part of a Ph.D. studentship funded by Fuse, the Centre for Translational Research in Public Health. KW, AI, LA, SB, and AB are members of Fuse, the Centre for Translational Research in Public Health (www.fuse.ac.uk). Fuse is a UK Clinical Research Collaboration (UKCRC) Public Health Research Centre of Excellence. Funding for Fuse from the British Heart Foundation, Cancer Research UK, National Institute of Health Research, Economic and Social Research Council, Medical Research Council, Health and Social Care Research and Development Office, Northern Ireland, National Institute for Social Care and Health Research (Welsh Assembly Government) and the Wellcome Trust, under the auspices of the UKCRC, is gratefully acknowledged. The views expressed in this paper do not necessarily represent those of the funders or UKCRC. The funders had no role in study design, data collection and analysis, decision to publish, or preparation of the manuscript.

## Conflict of Interest

The authors declare that the research was conducted in the absence of any commercial or financial relationships that could be construed as a potential conflict of interest.

## Publisher's Note

All claims expressed in this article are solely those of the authors and do not necessarily represent those of their affiliated organizations, or those of the publisher, the editors and the reviewers. Any product that may be evaluated in this article, or claim that may be made by its manufacturer, is not guaranteed or endorsed by the publisher.
