# Supplementary material for: Process Evaluation of Project FFAB (Fun Fast Activity Blasts): A Multi-Activity School-Based High-Intensity Interval Training Intervention
Source: Front Sports Act Living. 2021 Sep 20;3:737900. doi: 10.3389/fspor.2021.737900 (PMC8488272; doi:10.3389/fspor.2021.737900)
Supplement: Supplementary file 1 [file Table_1.DOCX]

Project FFAB qualitative process evaluation: Focus group script

(Icebreaker): In one sentence, how would you describe Project FFAB to your friends and family?

Project FFAB sessions

1. Discuss/describe the activities that you did.
2. Discuss/describe what you were asked to do during each session.
3. How did the activities make you feel?
4. How could you tell how hard you were working?
5. How did you know if you were doing well or not so well?
6. Did this influence your performance?

Session structure

1. What did you think about the way the sessions were structured?
2. Did the lunch time/ after schools sessions differ from the PE class ones?
3. Why did you attend sessions and why did you miss sessions?
4. When do you think is the best time to hold the extra sessions?
5. What did you think about the different people who led Project FFAB?

Future implementation and overall experience of the trial

1. Would you change anything about Project FFAB? If so, what?
2. How would you feel if something like this was carried on as part of PE (for example for a PE block) or as something you could do outside of school (for example at a leisure centre or after school).
3. Would the project be something you would recommend to friends and family and if so, for what reasons. If not, why not?
4. Do you feel any different after taking part in Project FFAB? If so in what ways?
5. What was your favourite/least favourite thing about Project FFAB?
6. How do you feel now that it is finished?
